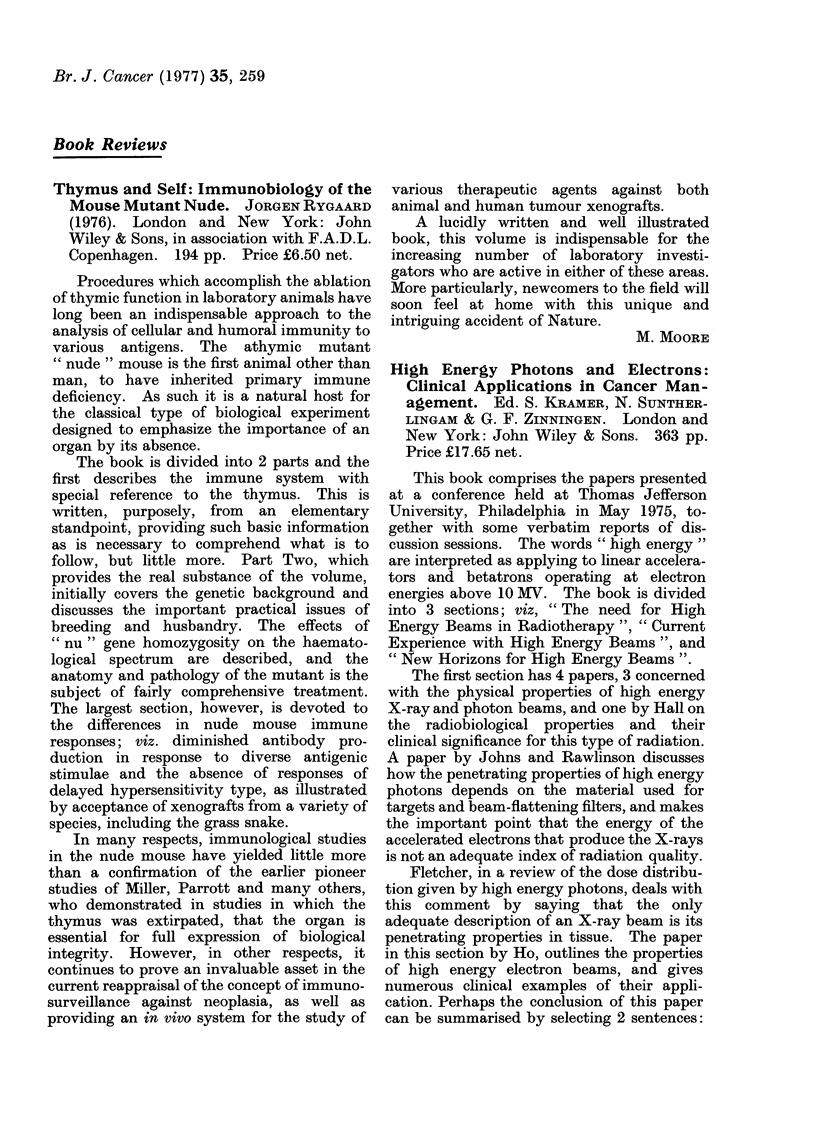# Thymus and Self: Immunobiology of the Mouse Mutant Nude

**Published:** 1977-02

**Authors:** M. Moore


*Br. J. Cancer* (1977) **35**, 259

### *Book Reviews*

**Thymus and Self: Immunobiology of the Mouse Mutant Nude.** JORGEN RYGAARD (1976). London and New York: John Wiley & Sons, in association with F.A.D.L. Copenhagen. 194 pp. Price £6.50 net.

Procedures which accomplish the ablation of thymic function in laboratory animals have long been an indispensable approach to the analysis of cellular and humoral immunity to various antigens. The athymic mutant " nude " mouse is the first animal other than man, to have inherited primary immune deficiency. As such it is a natural host for the classical type of biological experiment designed to emphasize the importance of an organ by its absence.

The book is divided into 2 parts and the first describes the immune system with special reference to the thymus. This is written, purposely, from an elementary standpoint, providing such basic information as is necessary to comprehend what is to follow, but little more. Part Two, which provides the real substance of the volume, initially covers the genetic background and discusses the important practical issues of breeding and husbandry. The effects of " nu " gene homozygosity on the haematological spectrum are described, and the anatomy and pathology of the mutant is the subject of fairly comprehensive treatment. The largest section, however, is devoted to the differences in nude mouse immune responses; *viz.* diminished antibody production in response to diverse antigenic stimulae and the absence of responses of delayed hypersensitivity type, as illustrated by acceptance of xenografts from a variety of species, including the grass snake.

In many respects, immunological studies in the nude mouse have yielded little more than a confirmation of the earlier pioneer studies of Miller, Parrott and many others, who demonstrated in studies in which the thymus was extirpated, that the organ is essential for full expression of biological integrity. However, in other respects, it continues to prove an invaluable asset in the current reappraisal of the concept of immunosurveillance against neoplasia, as well as providing an *in vivo* system for the study of various therapeutic agents against both animal and human tumour xenografts.

A lucidly written and well illustrated book, this volume is indispensable for the increasing number of laboratory investigators who are active in either of these areas. More particularly, newcomers to the field will soon feel at home with this unique and intriguing accident of Nature.

M. MOORE